# Is it Safe for Patients with Left Ventricular Assist Devices to Undergo Non-Cardiac Surgery?

**DOI:** 10.3390/medicina56090424

**Published:** 2020-08-23

**Authors:** Rafal Berger, Attila Nemeth, Christoph Salewski, Rodrigo Sandoval Boburg, Metesh Acharya, Alexander Weymann, Konstantin Zhigalov, Bastian Schmack, Michel Pompeu B. O. Sá, Christian Schlensak, Aron-Frederik Popov

**Affiliations:** 1 Department of Thoracic and Cardiovascular Surgery, University Hospital of Tübingen, 72070 Tübingen, Germany; attila.nemeth@med.uni-tuebingen.de (A.N.); christoph.salewski@med.uni-tuebingen.de (C.S.); rodrigo.sandoval-boburg@med.uni-tuebingen.de (R.S.B.); christian.schlensak@med.uni-tuebingen.de (C.S.); aron-frederik.popov@med.uni-tuebingen.de (A.-F.P.); 2 Department of Cardiac Surgery, Glenfield Hospital, Leicester LE3 9QP, UK; metesh.acharya@doctors.org.uk; 3 Department of Thoracic and Cardiovascular Surgery, West German Heart and Vascular Center Essen, University Hospital of Essen, University Duisburg-Essen, 45147 Essen, Germany; weymann.alexander@googlemail.com (A.W.); konstantin.zhigalov@yahoo.com (K.Z.); Bastian.Schmack@uk-essen.de (B.S.); 4 Department of Cardiovascular Surgery at the Pronto Socorro Cardiológico de Pernambuco (PROCAPE), 74970-240 Recife, PE, Brazil; michel_pompeu@yahoo.com.br

**Keywords:** left ventricular assist device, LVAD, chronic heart failure, non-cardiac surgery

## Abstract

*Background and Objectives:* Since the first use of ventricular assist devices (VADs) as bridge to recovery and bridge to cardiac transplantation in the early 1990s, significant technological advances have transformed VAD implantation into a routine destination therapy. With improved survival, many patients present for cardiac surgery for conditions not directly related to their permanent mechanical circulatory support. The aim of this study was to analyze the indications and outcomes of non-cardiac surgeries (NCSs) of left ventricular assist device (LVAD) patients in tertiary center. *Material and Methods:* We present a single-center experience after 151 LVAD implantations in 138 consecutive patients between 2012–2019 who had to undergo NCS during a follow-up period of 37 +/− 23.4 months on left ventricular assist device (LVAD). *Results:* A total of 105 procedures was performed in 63 LVAD recipients, resulting in peri-operative mortality of 3.8%. Twenty-five (39.7%) of patients underwent multiple surgeries. We found no significant difference in cumulative survival associated with the performed surgical interventions (*p* = 0.469). *Conclusion:* We demonstrated good overall clinical outcomes in LVAD patients undergoing NCS. With acceptable peri-operative mortality, NCS can be safely performed in LVAD patients on long-term support.

## 1. Introduction

Ventricular assist devices (VADs, Table 1) were originally introduced in 1990s as bridge to recovery and bridge to cardiac transplantation [1]. As a consequence of technological advances, left ventricular assist devices (LVADs) are now commonly utilized in patients with chronic left heart failure refractory to maximal medical management as destination therapy [2]. LVADs have evolved from pulsatile and extracorporeal devices to those implanted within the body to support the circulation with continuous flow provided by an axial (HeartMate II, Abbot, Chicago, IL, USA) or centrifugal (HeartWare HVAD, Medtronic, Dublin, Ireland and HeartMate III, Abbot, Chicago, IL, USA) pump [1]. Owing to the small size and high durability of the system, LVAD patients nowadays achieve many years of follow-up in the destination-therapy population [3].

With improved survival, there is an increasing number of LVAD recipients living as outpatients on long-term mechanical circulatory support. The latest trials report that one-year survival has increased to over 80% [4,5,6]. Moreover, the approval of LVADs for destination therapy in non-transplant candidates further increased the number of implantations [5,6,7]. As a result of improved reliability, quality of life, and mortality, patients supported with LVADs may develop a variety of non-cardiac conditions during follow-up, some of which require non-cardiac surgeries (NCSs) [8]. The peri-operative care of LVAD patients requiring NCS presents unique concerns regarding management of their clinical condition and maintenance of the implanted pump. Table 2 shows the various complications that can arise in the peri-operative management of LVAD patients [8,9].

Bleeding, especially within the gastro-intestinal (GI) tract, is a particular problem in LVAD recipients, with incidence of 14–31% [1]. There are many factors contributing to bleeding complications, including the need for anticoagulation, activation of fibrinolysis, loss of platelet number and function, mucosal ischemia, development of acquired von Willebrand’s syndrome, and development of arterial-venous malformation in the GI tract [1,10]. Similar to aortic stenosis, patients with continuous flow mechanical circulatory support (MCS) develop a state of high vascular shear stress, resulting in destruction of large multimers that play an import role in hemostasis.

Our study reports the indications, peri-operative management, and outcomes of a large population of LVAD patients undergoing NCS in our tertiary center.

## 2. Materials and Methods

All patients receiving LVAD at our institution from January 2012 to December 2019 were identified using our prospective LVAD database. Patients younger than 18 years were excluded from the analysis. Medical records of all patients were analyzed retrospectively for performed surgical procedures. We did not include patients who had NCS during their admission for LVAD implantation. In addition, LVAD replacement (*n* = 13) and LVAD explantation (*n* = 4) were excluded from the study. In the case of wound infections requiring open wound therapy and recurrent operative revisions, only the first intervention was taken into consideration. Furthermore, any operative intervention to treat post-operative complication was counted as complication rather than a separate procedure. Diagnostic procedures such as cardiac catheterizations and electrophysiologic examinations as well as endoscopies were not included in the study.

The primary outcome of interest was in-hospital mortality. Secondary outcomes included bleeding with requirement for blood transfusion, acute kidney injury, ischemic stroke, sepsis, and length of stay. Admissions for surgery were categorized as urgent and elective.

Approval for this study was obtained by the institutional review board of University of Tübingen with decision No. 194/2020BO2 on 15 April 2020.

### Statistical Analysis

All data analysis was performed using SPSS Statistics v26 (IBM, Armonk, NY, USA) software. Continuous variables are presented as mean +/− standard deviation (SD) or median, and categorical variables are reported as percentages of the total number of data points available. Mann–Whitney U testing was performed to compare variables between those LVAD recipients who did and did not undergo an NCS during the study period. Kaplan–Meier analysis was used to estimate survival. A *p* value less than 0.05 was considered significant.

## 3. Results

### 3.1. Patients’ Characteristics

A total of 151 LVAD implantations in 138 patients were performed in our center in the study period with 113 (81.88%) patients surviving to hospital discharge. Mean duration of follow-up was 37 +/− 23.4 (range 5–97) months, with patient number one being still alive and on LVAD support over eight years after implantation. In seven cases, details of post-operative follow-up was not available. Three durable LVAD models were encountered in the study population: Heartmate 2 (St Jude Medical, St Paul, MN, USA), Heart Mate 3 (St Jude Medical), and Heartware HVAD (Heartware, Framingham, MA, USA). The characteristics of these assist devices with basic parameters and number of implantations are presented in Table 3 [9]. Among 106 patients followed up, an NCS had to be performed in 63 (59.4%). We found no significant difference in patient sex, age at implantation, and type of device used. Cumulative observation time varied significantly between both groups. The characteristics of the patient population are shown in Table 4.

### 3.2. Surgical Procedures

A total of 105 surgical procedures were performed on 63 LVAD patients during follow-up. In 41 (39%) cases the surgery was performed on an urgent basis, and an elective intervention was performed in 61 (64%). Five (4.8%) cases underwent an ambulatory operation without anesthesia and discharge on the same day. One hundred (55.2%) patients required general anesthesia for surgery and admission to the cardiac surgery unit post-operatively. The types of procedures performed are shown in Table 5.

**Figure 1:**
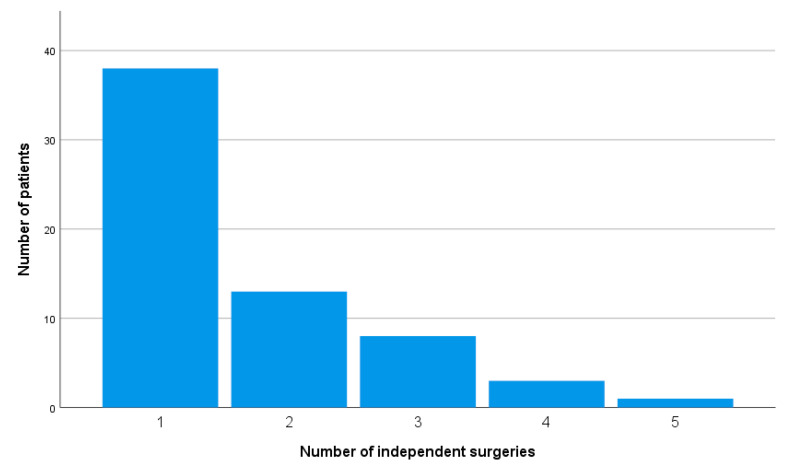
Number of non-cardiac surgeries per patient in an LVAD population.

LVAD-specific surgeries including late wound inspection and driveline complications dominated and comprised 24 (22.9%) of the total number of procedures. While on LVAD support, 25 (39.7%) patients required multiple surgeries, with one patient undergoing five separate interventions. Figure 1 shows the frequency of procedures in the LVAD patient population.

### 3.3. Management of Anticoagulation

In patients admitted for elective surgery, anticoagulation with warfarin was discontinued on admission and heparin infusion was commenced with a target partial thromboplastin time (PTT) of 50–70 seconds when the International Normalized Ratio (INR) had fallen below 2. The heparin infusion was discontinued approximately 4 h before the incision and restarted 4 h after surgery. For minor ambulant procedures (including pulse generator box replacement), oral anticoagulation therapy was continued with a target INR range of 2–2.5. In emergency situations, anticoagulation reversal with vitamin K administration was used when possible, and hemostatic agents such as fresh frozen plasma (FFP) and prothrombin complex concentrate (PCC) were utilized intra-operatively.

The benefit of antiplatelet therapy in LVAD patients has been demonstrated previously in [10]. At our center, we support the continuation of low-dose antiplatelet agents through the peri-operative period for NCS.

### 3.4. Survival

We reported four deaths in 105 procedures during the first 30 post-operative days, resulting in a peri-operative mortality of 3.8%. All four patients underwent emergency surgery. There were two neurosurgical procedures with craniotomy to evacuate intracranial hemorrhage, one major amputation following acute embolic limb ischemia, and one late wound infection, resulting in septicemia and multi-organ failure.

**Figure 2:**
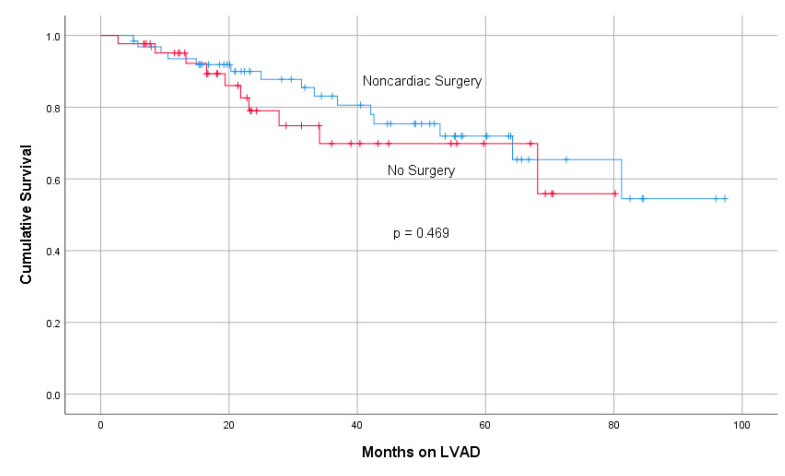
Kaplan-Meier survival curves in patients with a left ventricular assist device (LVAD) undergoing a non-cardiac surgery compared to those without surgery during follow-up. The p value was assessed by log rank analysis between both groups.

Figure 2 shows Kaplan-Meier survival curves for LVAD patients implanted in our center undergoing an NCS. For 63 LVAD recipients who underwent an NCS, a mean follow-up of 41.2 +/− 24 months with 15 (23.8%) deaths was recorded. Compared to 43 LVAD patients who did not undergo a concomitant surgery while on MCS, there was no significant worsening of cumulative survival despite undergoing an NCS.

## 4. Discussion

The application of LVADs had significantly reduced the morbidity and mortality of patients with end-stage heart failure [5,13]. With improved device reliability and durability over the last 20 years, a growing group of LVAD recipients require NCS on follow-up [13]. As shown by Briasoulis et al., up to 10% of all admissions of LVAD recipients are associated with a need for surgical intervention [8]. Moreover, the absolute number of NCS is increasing in this population as the life expectancy of patients with advanced heart failure treated with LVAD continues to rise [3,14]. Our analysis showed that follow-up time alone is a significant factor for NCS.

Although studies regarding surgeries on patients with MCS are already available, many of them evaluate patients using various assist devices and in early phase immediately after implantation. In our analysis, we focused on surviving patients who were discharged with modern continuous flow LVADs and in stable medical condition that made it possible for ambulatory aftercare.

Infection, bleeding, and thrombo-embolism represent the most prevalent complications in patients with implanted LVADs within the community [15], and require careful consideration as they may continue to pose a clinical issue within the peri-operative period. While device malfunction is a rarer phenomenon, almost a quarter of NCSs performed in our series were related specifically to the LVAD, to include surgical management of driveline infection and wound inspection under general anesthesia.

Owing to the risk of thromboembolic complications, patients with LVADs are usually anticoagulated and receiving aspirin [13]. To reduce bleeding risks, precise management of coagulation is essential to preventing pump thrombosis and thromboembolic complications. Nevertheless, peri-operative bleeding is a common complication during NCS and the need for blood transfusion in our group of patients is similar to that reported in other studies [13]. Significant variability exists in the literature regarding the timing of post-operative resumption of anticoagulation in LVAD patients undergoing NCS. In the present series, heparin infusion was recommenced at 4 h following surgery or when there were no further concerns regarding bleeding risk, and transitioned to warfarin once INR exceeded 2. We did not observe any major bleeding complications in patients undergoing elective NCS.

Surgical procedures for LVAD-specific complications such as wound inspections and driveline revisions dominated the analysis and were responsible for 22.9% of NCS procedures in our study. Despite good clinical outcomes, such procedures are also necessary after many years of reliable LVAD support.

We reported a 30-day post-operative mortality of 3.8%, similar to that observed in previous reports (range 0–16.7%) [3,5]. The four patients who died had undergone emergency surgery, including neurosurgery for evacuation of intra-cranial hemorrhage in two patients, major amputation for limb ischemia in one patient, and surgery for wound infection in one patient who later developed multi-organ failure from sepsis. It is likely that mortality could be attributed to the severity of their presenting pathology and the resultant systemic complications, and not directly linked to complications arising from LVAD support. However, the requirement for continued anticoagulation, in particular, and associated bleeding complications, could have had a negative effect on post-operative outcomes in those undergoing emergency procedures, compared to elective NCS.

The cumulative survival of LVAD recipients undergoing an NCS does not differ significantly to that in the group not undergoing a surgical procedure, as noted by Bhat et al [3]. Discontinuation of anticoagulation before non-cardiac surgery is safe but requires clinical staff to be familiar with the LVAD to prevent LVAD thrombosis and systemic thromboembolism.

Since the majority of NCS in patients with LVADs are performed by non-cardiothoracic surgeons, a multi-disciplinary approach is essential to ensure safety in this complex patient group. International guidelines advise that a cardiovascular surgeon should be informed prior to planned NCS and should be immediately available for consultation [16]. Similarly, a non-cardiac anesthesiologist can care for stable patients on LVADs undergoing NCS, but a dedicated cardiac anesthesiologist is recommended for those patients displaying hemodynamic variability or with significant additional comorbidities [17]. Important anesthetic considerations include optimizing right ventricular function and preload, maintaining LVAD pump speed, and management of systemic vascular resistance [17,18]. In many circumstances, however, intra-operative anesthetic management in patients with an implanted LVAD undergoing NCS is not significantly different to that in patients without an LVAD. Ideally, NCS in these patients should be conducted at a cardiothoracic transplant center, where expertise in LVAD management is readily available. Alternatively, patients can be transferred post-operatively to a VAD center for continued observation prior to discharge home. At our institution, although NCS may be carried out in non-cardiac units within the same hospital, close collaboration with the cardiac unit is facilitated by its clinical and logistical staff. A multidisciplinary approach within a tertiary center with availability of LVAD technicians and perfusionists during surgery, provision of peri-operative care by cardiac anesthesiologists, and post-operative care by nursing stuff and intensivists familiar with LVADs are important factors that impact the post-operative outcomes of patients with LVADs undergoing NCS [8,9,19,20].

As a limitation of our study, its retrospective nature carries the risk of selection bias. As characteristic to retrospective reviews, the available data were limited to those parameters recorded for clinical purposes. Furthermore, any patients with LVADs unknowingly admitted to external institutions for NCS during follow-up could not be identified and, thus, were not analyzed.

Despite these limitations, our study highlights that surgical intervention can be performed safely in this demanding group of patients. Our findings are supported by those of existing studies investigating the outcomes of NCS while on mechanical circulatory support [2,3,8,13,18,21]. The prevalence of NCS in LVAD recipients is still increasing, with a growing demand for management of LVAD recipients outside the implanting center and without access to a cardiac surgical team on standby. As shown in Table 5, with appropriate planning an LVAD patient can undergo a broad range of surgeries with a good outcome [22].

## 5. Conclusions

Based on the results of our single-center experience, non-cardiac surgery can be performed on LVAD recipients with acceptable mortality. Further standardized and transparent evaluation of LVAD patients undergoing NCS, especially in a multi-institutional context, is essential to maintain a high standard of care in this demanding and growing population.

## Figures and Tables

**Table 1:** Abbreviations.

GI	Gastrointestinal
INR	International Normalized Ratio
LVAD	Left Ventricular Assist Device
MCS	Mechanical Circulatory Support
NCS	Non-Cardiac Surgery
VAD	Ventricular Assist Device
VATS	Video-Assisted Thoracoscopic Surgery

**Table 2:** Complications associated with VADs [1,11].

Hypotension Right heart failure Bleeding Infection Thromboembolism Thrombosis of the device GI bleeding Arrhythmias Thoracic effusion Neurologic complications Thrombosis or fusion of aortic valve Renal failure Device failure Hemolysis

Abbreviations: VAD, ventricular assist device; GI, gastrointestinal.

**Table 3:** Characteristics of implanted LVADs (*n* = 106).

Parameter	Heartmate 2	Heartmate 3	HVAD
Ongoing patients, n (%)	47 (44.3)	28 (29.4)	31 (29.2)
Speed, rpm	8000–10,000	5000–6000	2400–3200
Flow, L/min	4–7	4–6	4–6
Power, W	5–8	4.6–6.5	3–7

Abbreviations: LVAD, left ventricular assist device; HVAD, HeartWare left ventricular assist device; Adapted from Kiamanesh et al. [12].

**Table 4:** Patients’ characteristics (*n* = 106).

	LVAD Patients with NCS	LVAD Patients without NCS	*p*-Value
Study population:			
Number of Patients, n (%)	63 (59.4)	43 (40.6)	
Male, n (%)	53 (84.1)	34 (79.1)	0.714
Age at implantation (years)	55.6 +/− 12.4	56.2 +/− 14.6	0.418
Age at surgery (years)	59.4 +/− 13	N/A	
Time on LVAD (months)	21.5 +/− 18.4	N/A	
Type of LVAD:			
Heart Mate II, n (%)	28 (44.4)	19 (44.2)	0.979
Heart Mate III, n (%)	20 (31.7)	8 (18.6)	0.314
Heartware HVAD, n (%)	15 (23.8)	16 (37.2)	0.318
Follow-up (months)	41.2 +/− 24	30.7 +/− 21.3	<0.05
Deaths, n (%)	15 (23.8)	10 (23.3)	0.948
NCS, n	105	N/A	
Urgent, n (%)	41 (39)	N/A	
Elective, n (%)	64 (61)	N/A	
30-day mortality, n (%)	4 (3.8)	N/A	

Abbreviations: LVAD, left ventricular assist device; HVAD, HeartWare left ventricular assist device; NCS, non-cardiac surgery; N/A, non applicable.

**Table 5:** Type of non-cardiac surgical procedures (*n* = 105).

Procedure	Number of Procedures
Abdominal Surgery: Cholecystectomy Femoral hernia repair Gastrectomy Gastric banding Ileostomy formation Adhesiolysis Vascular Surgery: AV Shunt formation Pseudoaneurysm repair Groin haematoma drainage Extremity haematoma drainage Urologic Surgery: Transurethral resection Neobladder reconstruction Transurethral cauterization for bleeding Circumcision Oral Surgery Neurologic Surgery: Craniotomy Cerebral shunt formation Spinal haematoma drainage Orthopaedic Surgery: Hip replacement Minor amputation Major amputation Joint empyema drainage Spinal fusion Thoracic Surgery: VATS, cardiac denervation Head and Neck Surgery: Tracheostomy formation Cauterization for epistaxis Basal cell carcinoma resection Driveline/Wound Surgery ICD/Pacemaker: Implantation Generator Box Replacement Wire Revision Loop excision for cervical cancer Ambulant Surgery: IV dialysis port insertion Pleural drainage	4 3 4 2 2 1 1 1 1 2 3 1 1 1 1 7 1 1 3 2 1 1 1 2 2 1 1 24 5 13 5 1 3 2

Abbreviations: VATS, Video Assisted Thoracoscopy; ICD, Implantable Cardioverter Defibrillator.
